# Chronic lymphocytic leukemia skin infiltrates presenting as eyebrow alopecia and erythematous pruritic papules refractory to treatment with topical steroids

**DOI:** 10.1002/ccr3.3184

**Published:** 2020-08-05

**Authors:** Jane L. Zhu, Heather W. Goff

**Affiliations:** ^1^ Department of Dermatology University of Texas Southwestern Medical Center Dallas Texas

**Keywords:** alopecia, chronic lymphocytic leukemia, eyebrow, leukemia cutis

## Abstract

We describe a patient who presented with erythematous papules and hair loss solely limited to the eyebrows. Clinicians should be aware of this presentation of leukemia cutis and consider this diagnosis in a patient with a history of CLL.

## Graphical abstract

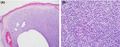

## 1 INTRODUCTION

Chronic lymphocytic leukemia (CLL) is a chronic lymphoproliferative disorder characterized by progressive accumulation of functionally incompetent mature lymphocytes. Up to 25% of patients with CLL develop leukemic skin infiltrations, referred to as leukemia cutis.^1^ We report a patient with CLL who developed leukemia cutis presenting as erythematous papules located on the eyebrows and eyebrow alopecia, clinically mimicking frontal fibrosing alopecia.

## 2 CASE PRESENTATION

A 56‐year‐old Caucasian female patient presented to the UT Southwestern outpatient dermatology clinic for an annual skin check. Her past medical history was notable for chronic lymphocytic leukemia (CLL) with Richter transformation; she had previously been treated with 6 cycles of rituximab, cyclophosphamide, doxorubicin, vincristine, prednisolone, and autologous stem cell transplantation with good response. Subsequently, she had slow progressive CLL without recurrence of large cell transformation controlled with ibrutinib, which caused cardiac issues, paresthesias, and GI distress, requiring dose reduction and ultimately discontinuation. At the time of her presentation, she had not been on any systemic therapies for one month. She had stable leukocytosis (60.73) and was being closely followed by hematology and oncology.

**FIGURE 1:**
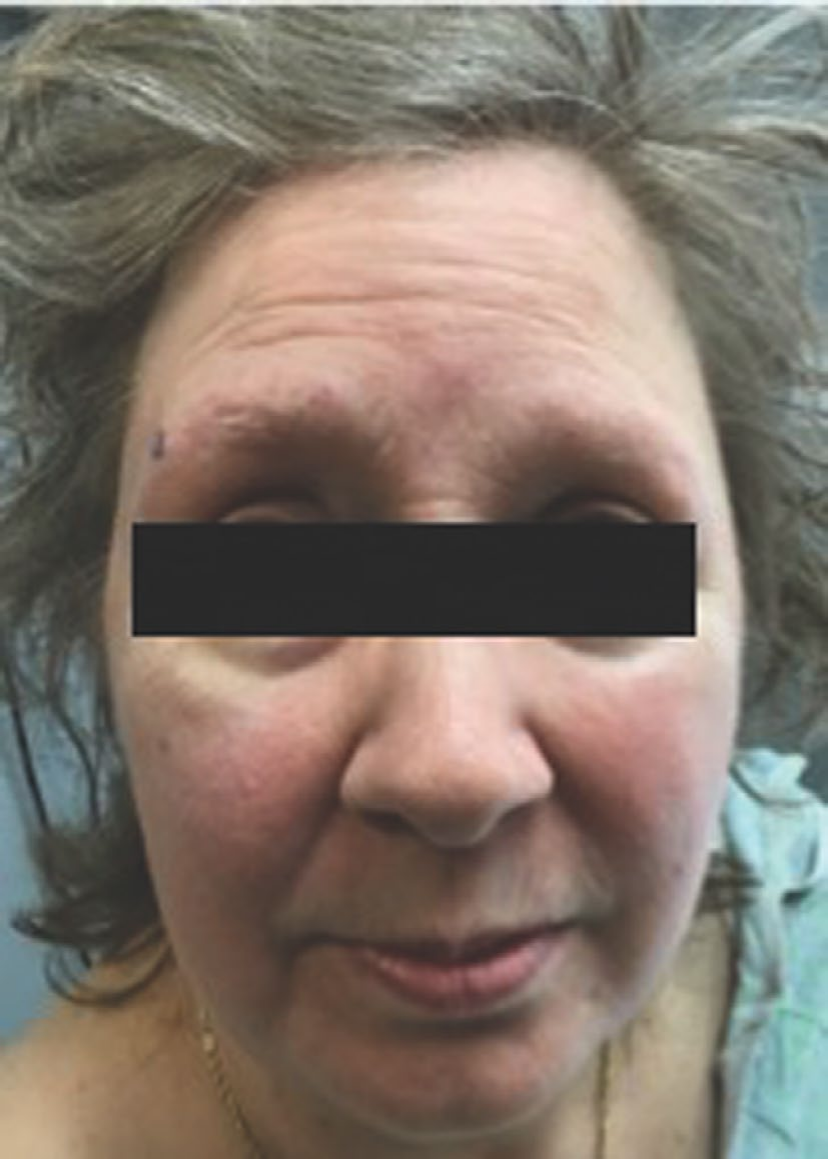
Patient with bilateral loss of eyebrows and perifollicular erythematous papules at time of initial presentation

Her dermatologic history consisted of melanoma in situ, basal cell carcinomas, and rosacea. During her visit, she reported erythema and irritation on her anterior neck and face with concomitant lateral eyebrow loss, which she attributed to rosacea. Her bilateral eyebrows were hypotrichotic with mild perifollicular erythema and plugging of follicles (Figure 1); her anterior neck had pityriasiform flat, pink to tan patches and macules. The frontal hairline and remainder of the scalp were clear. A clinical diagnosis of lichen planopilaris was made, and she was prescribed 2.5% hydrocortisone cream. She was also started on 15% azelaic acid cream and metronidazole 1% gel for rosacea. She returned to clinic six months later with continued loss of eyebrows bilaterally and scaling. At that visit, she was thought to have lichen planopilaris admixed with seborrheic dermatitis. As such, ketoconazole cream was added to her treatment regimen.

At her next few follow‐up visits, she continued to have issues with her rosacea, reporting redness and loss of eyebrows bilaterally despite treatment with topical steroids and ketoconazole cream. She denied having any burning in the affected areas or hair loss in other areas of the body. On examination, perifollicular erythematous papules with follicular plugging on bilateral temples and bilateral eyebrows were noted. The frontal hairline and remainder of the scalp were clear. Other diagnoses such as ulerythema ophryogenes and frontal fibrosing alopecia were considered. With these new examination findings and her persistent symptoms, a punch biopsy was taken from the right temple, which showed slight spongiotic dermatitis. Given these biopsy results, she was continued on hydrocortisone cream.

**FIGURE 2:**
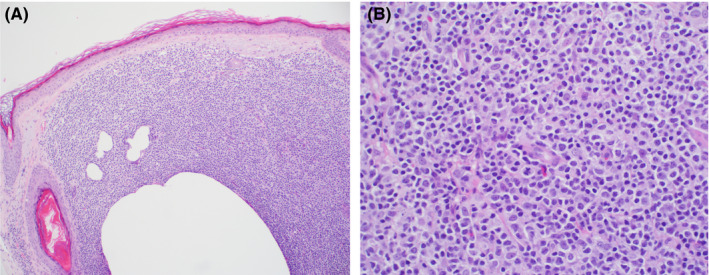
Histology of patient's punch biopsy reveals chronic lymphocytic leukemia. (A) Lymphocytic nodular infiltrates in superficial dermis (Hematoxylin and eosin, 100×), (B) Infiltrates were composed of small atypical lymphocytes with round to slightly irregular nuclei and condensed chromatin (Hematoxylin and eosin, 400×)

At the following visit, she continued to experience moderate pruritus and eyebrow loss, which had not improved at all since her last visit. Consequently, a punch biopsy was taken from the right eyebrow, which revealed several nodular infiltrates in the superficial dermis with small atypical lymphocytes with round to slightly irregular nuclei and condensed chromatin (Figure 2). The atypical lymphocytes were positive for CD5, CD20, PAX5, BCL2, and MUM‐1. As such, the histopathology findings were consistent with cutaneous involvement by the patient's previously diagnosed chronic lymphocytic leukemia/small lymphocytic lymphoma. No morphologic features of large cell (Richter's) transformation were identified.

As a result of these findings, the patient was started on acalabrutinib. Her itching and discomfort resolved almost immediately, and the erythematous papules began to fade within a few weeks of chemotherapy initiation.

## 3 DISCUSSION

Leukemia cutis is thought to be caused by seeding of the malignant cells and typically presents in patients who have already been diagnosed with leukemia. Cutaneous manifestations of leukemia such as leukemia cutis less frequently occurs simultaneously with systemic disease.^2^, ^3^ Generally, CLL patients presenting with skin infiltrates have a good prognosis and local therapy for skin disease can delay or avoid the need for systemic treatment of CLL.^4^ However, prognosis is poor in those who have leukemic cells that demonstrate a Richter's transformation or when skin infiltrates develop after the diagnosis of CLL.^2^, ^3^, ^5^, ^6^ Chronic lymphocytic leukemia has previously been reported to cause chronic and relapsing pruritic skin lesions^7^ as well as indurated plaques of the eyebrow.^8^ Additionally, Hay et al reported a case of a 63‐year‐old male who developed swelling and redness of the ears, nose, and eyebrows 10 years following a diagnosis of CLL. The CLL infiltrate was interestingly limited to hairy parts of the scalp and eyebrows and prominent parts of the face.^9^

The differential diagnosis for eyebrow alopecia also includes ulerythema ophryogenes and frontal fibrosing alopecia. Ulerythema ophryogenes is a disorder characterized by inflammatory keratotic papules on the lateral aspects of the eyebrow that may result in scars and alopecia. It has been reported to occur in patients with congenital anomalies,^10^ Cornelia de Lange syndrome,^11^ and Noonan syndrome.^12^ However, its onset is more common at earlier ages. Frontal fibrosing alopecia is a condition that mostly affects postmenopausal women, and eyebrow hair loss has been reported to be the first sign of FFA in up to 48% of patients.^13^ However, it can be distinguished from other causes of eyebrow alopecia as it has been reported to selectively affect the intermediate and the vellus‐like follicles of the frontal margin and eyebrows.^14^

Our patient presented with erythematous papules and hair loss solely limited to the eyebrows with minimal perifollicular erythema in the temporal scalp without involvement of other regions. A similar presentation has not been previously reported. Clinicians and providers should be aware of this presentation of leukemia cutis and consider this diagnosis in a patient with a history of CLL.

## CONFLICT OF INTEREST

The authors have no conflicts of interest to disclose.

## AUTHOR CONTRIBUTIONS

JZ: involved in conceptualization, drafting of original manuscript, and revising and editing of manuscript. HG: involved in conceptualization, drafting of original manuscript, revising and editing of manuscript, and supervision.

## ETHICAL APPROVAL

The authors are accountable for all aspects of the work in ensuring that questions related to the accuracy or integrity of any part of the work are appropriately investigated and resolved.
